# Microarray testing in clinical diagnosis: an analysis of 5,300 New Zealand patients

**DOI:** 10.1186/s13039-016-0237-9

**Published:** 2016-03-31

**Authors:** Adrian Mc Cormack, Karen Claxton, Fern Ashton, Philip Asquith, Edward Atack, Roberto Mazzaschi, Paula Moverley, Rachel O’Connor, Methat Qorri, Karen Sheath, Donald R. Love, Alice M. George

**Affiliations:** Diagnostic Genetics, LabPLUS, Auckland City Hospital, PO Box 110031, Auckland, 1148 New Zealand; Present address: Pacific Edge Ltd, 87 St David St, North Dunedin, 9016 New Zealand

## Abstract

**Background:**

The use of Microarray (array CGH) analysis has become a widely accepted front-line test replacing G banded chromosome studies for patients with an unexplained phenotype. We detail our findings of over 5300 cases.

**Results:**

Of 5369 pre and postnatal samples, copy number variants (CNVs) were detected in 28.3 %, of which ~40 % were deletions and ~60 % were duplications. 96.8 % of cases with a CNV <5 Mb would not have been detected by G banding. At least 4.9 % were determined to meet the minimum criteria for a known syndrome. Chromosome 17 provided the greatest proportion of pathogenic CNVs with 65 % classified as (likely) pathogenic. X chromosome CNVs were the most commonly detected accounting for 4.2 % of cases, 0.7 % of these being classified as cryptic (likely) pathogenic CNVs.

**Conclusions:**

Microarray analysis as a primary testing strategy has led to a significant increase in the detection of CNVs (~29 % overall), with ~9 % carrying pathogenic CNVs and one syndromic case identified per 20 referred patients. We suggest these frequencies are consistent with other heterogeneous studies. Conversely, (likely) pathogenic X chromosome CNVs appear to be greater compared with previous studies.

## Background

The molecular karyotype (microarray) is now recognised as a first tier diagnostic test for patients with wide-ranging phenotypes and has led to greater sensitivity in the detection of sub-microscopic genomic changes, largely replacing microscopy for constitutional analysis in many laboratories. Reported findings have varied due mainly to sample numbers, different platforms, variation in probe coverage and also analysis and reporting guidelines. Overall, as the technology has developed, it has aided in the diagnosis of pathogenic copy number variants (CNVs) and also genotype-phenotype correlations.

Published reports tend to fall into three groups. First, those based on sample sizes such as Park et al. [1] who arrayed 407 peripheral bloods, and found an 8.3 % pathogenic rate. Conversely, much larger scale array sample numbers have included Shaffer et al. [2] who reported on 8800 patients with an overall CNV yield of ~12 %, and Ahn et al. [3] who reported on a similar-sized sample number with an overall CNV yield of ~25 %. Secondly, those focused on specific chromosomes such as Willemsen at al. [4] who reported on X chromosome CNV studies and found a pathogenic frequency of 0.3 %. Finally, small cohorts of patients with specific phenotypes, such as those described by Coutton et al. [5] and D’Arrigo et al. [6] who studied children with varying degrees of intellectual disability. These workers reported an overall CNV yield of ~30 % and pathogenic yield of 16–21 %. We report the microarray analysis of over 5300 New Zealand patients from a heterogeneous population of postnatal bloods, detail our findings, outline some interesting case series, and compare our diagnostic yields to those reported by others.

## Methods

### Population & samples

The laboratory, based at Auckland City Hospital, serves an area of the North Island of New Zealand with a population of approximately 1.7 million. The demographics include a diverse ethnic mix including European Caucasian, and a large Asia-Pacific population including indigenous Maori, Pacific islanders & Han Chinese. Patients were referred from a variety of centres including paediatric, neonatal, outreach clinics and in-house clinical geneticists. The age of patients were from newborn upwards, with referral reasons ranging from single or multiple congenital abnormalities to neurodevelopmental delay with or without neuropsychiatric disorders. From late 2014, following national agreement, the laboratory expanded the service to include prenatal testing on patients with two or more abnormalities detected on ultrasound examination. A total of 5369 samples were analysed up to the end of June 2015 including 230 products of conception (POC’s) and 40 prenatal samples. The patients, or parents in the case of neonates, provided informed consent for diagnostic testing; the New Zealand Health and Disability multi-region Ethics Committee has ruled that cases of patient management do not require formal ethics committee approval.

### Array system details & methodology

Patient DNAs were screened for CNVs using either the Affymetrix® Cytogenetics Whole-Genome 2.7 M Array or the CytoScan® 750K Array. The former comprises 2.36M non-polymorphic markers and 400 k SNP markers with an average probe spacing of 1 kb, and the latter comprises 550 k non-polymorphic markers and 200 k SNP markers, with an average probe spacing of 4.1 kb. Practical procedures were carried out according to the manufacturer’s instructions. In the case of the 2.7M array, this entailed whole genome amplification of 100 ng gDNA followed by purification. The purified DNA was then fragmented, labelled and hybridised overnight onto an array. The arrays were washed using an Affymetrix® GeneChip® Fluidics Station, then scanned using an Affymetrix® GeneChip® scanner. In the case of the 750 K array, 250 ng gDNA was digested with *Nsp*1 and then ligated to a common oligonucleotide adaptor for amplification by PCR. After purification, the PCR products were fragmented and then labelled with a biotinylated deoxynucleotide analogue using the TdT enzyme followed by overnight hybridisation to the array. The arrays were washed using an Affymetrix® GeneChip® Fluidics Station (which included DNA-selective staining with a streptadivin conjugated reporter molecule) and then scanned using an Affymetrix® GeneChip® scanner. The data files generated for each sample were analysed using Chromosome Analysis Suite (ChAS) Software (v1.0.1 or v1.2.2). All results were subject to quality control for SNPQC, MAPD, waviness, sex, and signal intensity. Samples which failed overall QC were repeated.

### CNV interpretation

The clinical relevance of each CNV was assessed using the guidelines described by the American College of Medical Genetics, the ClinGen Structural Variant Working Group (formerly the International Standards for Cytogenomic Arrays Consortium) and the Australasian Society of Diagnostic Genomics [7–9]. Primarily our approach followed the evidence-based review process outlined by Riggs et al. [8] and involved the assessment of published data for, or against, dosage sensitivity of the genes within each CNV. These data often included genotype/phenotype correlations of previously documented cases, *in vitro* functional data and pedigree segregation data. The outcomes from these searches were added to data from publicly available curated CNV databases and sequence mutation databases for healthy or disease cohorts (DECIPHER database, Database of Genomic Variants (DGV), Human Gene Mutation Database). This information was used to classify each region based on its expected clinical significance (benign, likely benign, unknown, uncertain, likely pathogenic, pathogenic).

Incidental findings are a category of variants presenting a unique set of challenges within the clinical environment [10] and are compounded by a lack of clarity of, or difference in, the local medical ethics framework in which the results are reported. There is currently an absence of agreement in the international community regarding best practice guidelines for reporting incidental or secondary findings in general [11]. Incidental findings were therefore handled on case-by-case basis in consultation with the clinician.

### Inheritance studies

Inheritance studies were primarily performed using BAC FISH probes for the CNV of interest or, very rarely, by microarray. Referral was made to our clinical genetic service for counselling prior to follow up studies being undertaken. In the case of more than one CNV being detected in a proband, those CNVs of interest for further investigation were selected by the clinical service. Only 237 cases were followed up, with confirmed *de novo* and inherited frequencies of 95 and 161, respectively. Prior to the advent of microarray technology insertional translocations were thought to be very rare, with an estimated occurrence of approximately 1/10,000 [12, 13]. Subsequently a frequency of 1/500 has been suggested [14].

## Results

The scale of CNVs ranged from a 146 kb deletion to a 57.5 Mb terminal duplication. The latter was a dup(3)(p23q29) detected in a male neonate with heart defects, clinodactyly and dysmorphic features. Conversely, the 146 kb deletion (18q21.3) was detected in a 16 year old presenting with epilepsy, intellectual disability and hyperventilation. This deletion contained part of the *TCF4* gene and is associated with Pitt-Hopkins syndrome (OMIM # 610954), which is consistent with the referral reason.

We detected only one insertional translocation: a 16 year old girl with a history of developmental delay including learning difficulties, dyspraxia and gross motor skills with a 2.1 Mb duplication of 9q34.3q34.3. Subsequent BAC FISH follow-up studies showed that this duplicated region was inserted into 19p13.4. Parental analysis showed that this was inherited from her phenotypically normal father who had a balanced insertional translocation.

**Fig. 1:**
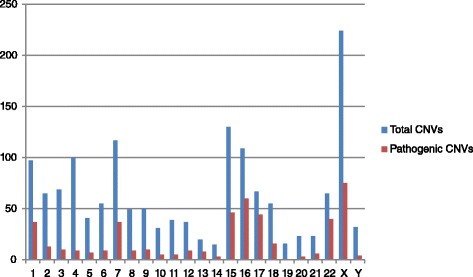
Graphical illustration of the total number of CNVs detected per chromosome (*Blue*) alongside the number of pathogenic CNVs detected per chromosome (*Red*)

Table 1, Fig. 1 and Table 2 document the overall CNV findings, total number of CNVs as well as pathogenic CNVs per chromosome, and the number of the most common genomic syndromes, respectively. Our overall reportable CNV detection frequency was 28.3 %, of which ~40 % were deletions and ~60 % were duplications. Aneuploidy (such as Down, Edwards’ and Kleinfelter syndrome) accounted for 0.7 % of cases. Initially samples were processed for array analysis irrespective of whether they were likely to be aneuploid. Subsequently, these samples were screened by FISH or G banding prior to being arrayed, which resulted in a reportable CNV detection frequency of 27.7 %. This adjusted figure is similar to the 25.2 % reported by Ahn et al. [3] who used an Agilent 4 × 44 oligonucleotide array platform (AMADID 017457), followed by an 8x60k platform (AMADID 028469), but significantly greater than Shaffer et al. [2] who reported ~12 % using an unspecified BAC array platform. Previous studies have shown that deletions are generally detected more frequently than duplications with the suggestion that duplications are associated with a milder phenotype and are therefore less likely to be referred [15]; however, at least one large scale study has shown a similar result to ours, albeit with a larger overall CNV duplication detection frequency compared to deletions [3]. Apart from chromosomal aneuploidy, approximately 171 cases (3.2 %) carried a CNV greater than 5 Mb in size and would be detected by G banding, while 96.8 % of cases would have been below G banding resolution.

**Table 1:** Overall findings of microarray testing

	Number	%
Total no of cases	5369	-
Abnormal	1523	28.3
Imbalances		
Deletions	652	12.1
Duplications	871	16.2
CNV >5 Mb	171	3.2
Mosaic	8	0.1
Pathogenic CNVs	462	8.6
Aneuploidy	37	0.7
Syndromes	262^a^	4.9
X Chromosome	75	1.4
Inheritance studies		
No of cases	237	4.4
*De novo*	95	1.8
Maternal	103	1.9
Paternal	58	1.0

^a^Also includes susceptibility loci/low penetrance

**Table 2:** Numbers of the most common genomic syndromes detected among 5369 New Zealand patients^a^

OMIM #	Syndrome	Number	del x1	dup x3	trp x4
612474/612475	1q21.1^c^	31	9	22	-
612530	1q41q42	3	3	-	-
612313	2q32	2	2	-	-
609425	3q29	4	4	-	-
194190	4p16.3	3	1	2	-
149730	LADD syndrome	2	2	-	-
123450	Cri du Chat	1	1	-	-
117550	Sotos	1	1	-	-
194050/609757	7q11.23	16	12	4	-
613729	Distal 7q11.23	4	4	-	-
610253	Kleefstra	2	2	-	-
194072	WAGR (*WT1*)	1	1	-	-
601803	Pallister-Killian	1	-	-	1
615656	15q11.2^c^	37	21	16	-
105830/176270	AS/PWS	10	10	-	-
612001	15q13.3^c^	17	17	-	-
611913/614671	16p11.2^c^	29	21	7	1
613444	16p11.2^c^	5	5	-	-
613604	16p12.2-p11.2^c^	1	1	-	-
Hanner (2009) [22]	16p13.11^c^	13	6	7	-
614527/614526	17q12 (*HNF1β*)	12	5	7	-
610883	Potocki-Lupski	7	-	7	-
182290	SMS	1	1	-	-
118220/162500	CMT/NHPP	6	5	1	-
247200/613215	17p13.3	5	3	1	1
610443/613533	17q21.31	5	3	2	-
613675	NF1	3	3	-	-
115470	Cat eye syndrome	1	-	1	-
188400/192430	DGS/VCFS	13	13	-	-
606232	Phelan McDermid	8	8	-	-
312865/400020	*SHOX* ^b^	3	3	-	-
300260	*MECP2*	2	-	2	-
310200	DMD	2	2	-	-

^a^ The table uses the format reported by Ahn et al. [3]

^b^ includes enhancer deletion of the *SHOX* gene

^c^ susceptibility loci/low penetrance

Eight hundred forty-two cases (15.7 %) were classified as having a variant of unknown significance (VOUS), with insufficient evidence to classify the CNV as pathogenic, as well as insufficient information in the DGV database to dismiss it as common within the normal population. Four hundred sixty-two cases (8.6 %) were classified as pathogenic. Approximately 45 % of the pathogenic CNVs were categorised as likely pathogenic; the majority met the minimum critical region (MCR) for a recognised syndrome with wide-ranging variable penetrance with or without minimal phenotypic information. Fifty-five percent of the pathogenic cases were described as pathogenic on the basis of aneuploidy, large deletions and structural rearrangements, known single gene haploinsufficiency, or a minimum critical region for a syndrome with well-established and published penetrance rates. 118 cases (2.1 %) were classified as unknown as there were no similar cases within the peer-reviewed literature and inadequate evidence in databases of normal variants to assist interpretation. Finally, approximately 101 reported CNVs (1.9 %) were classified as unlikely to be pathogenic. Most of these CNVs comprised chromosomal regions that were gene-poor and contained mainly non-coding reference sequences with a complex phenotype.

## Discussion

Previous postnatal studies have indicated that array CGH and cytogenetic analysis detected pathogenic chromosome abnormalities in ~7 % of children referred for testing [2]. Our results indicate a pathogenic frequency of 8.6 % for postnatal cases. The small number of prenatal and products of conception cases (4.5 %) would not have significantly influenced the abnormality frequency (only 12 pathogenic), which consisted almost exclusively of postnatal peripheral blood samples. The frequency of 8.6 % compares to 6.9 % reported by Shaffer et al. [2], and 8.3 and 9.8 % reported by Park et al. [1] (who used a customised whole genome BAC array) and Ahn et al. [3]), respectively. The guidelines used by each of these groups for classification of the CNVs were not specified.

We estimate that at least 262 of our pathogenic cases (4.9 %) were consistent with a known syndrome (including susceptibility loci or low penetrance syndromes) giving a syndrome detection frequency of one per 20 cases, which is the same as that reported by Ahn et al. [3]. A significant proportion of these cases were confined to a small number of chromosomes with 65 % (44) of CNVs detected on chromosome 17 classified as pathogenic (Fig. 1). This chromosome is predisposed to genomic rearrangements having a large number of dosage-sensitive genes, together with many interspersed nucleotide elements (LINEs/SINEs) and segmental duplications (SDs) [16]. The other predominant chromosomes carrying pathogenic CNVs were chromosomes 22 which had 61 % of CNVs detected classified as pathogenic, followed by chromosomes 16 (55 %), 1 (38 %), 15 (35 %), X (33 %), and 7 (31 %). Despite having a large percentage of CNVs and also increased genomic size, chromosome 4 CNVs were largely classified as VOUS, and only nine were pathogenic.

The most common confirmed syndrome was 1q21.1 duplication syndrome(s), which included both the smaller proximal (TAR syndrome) and larger proximal and distal duplication syndrome regions (Table 2). The most commonly detected CNV occurred at 15q11.2, which contains the *TUBGCP5* [*GCP5*], *CYFIP1*, *NIPA2 & NIPA1* genes. Deletions of this region have variable penetrance, and are associated with developmental and language delay, neurobehavioural disturbances and psychiatric problems; the corresponding micro-duplication of this region has been suggested to be a risk factor for some neurobehavioral disorders [17]. Susceptibility loci and low penetrance syndromes such as these make up 2.5 % of our detected CNVs.

4.9 % of reported CNVs involved the sex chromosomes with the majority being on chromosome X and only 2 cases involving the Y chromosome being classified as pathogenic. The Affymetrix platform lacks coverage of the *SRY* gene, meaning that cryptic abnormalities involving this cannot be detected. Variants of the X chromosome form a subset due to dosage and also inheritance differences between the sexes. Approximately 53 % (119) of X chromosome CNVs (224) were classified as VOUS, and 33 % (75) as (likely) pathogenic. Only 5 % (11) of X chromosome CNVs were classified as unlikely to be pathogenic, and almost exclusively were small CNVs in females. Of the cryptic X chromosome CNVs classified as VOUS, four cases (male) were found to be partial *AFF2* (FRAXE) gene duplications ranging from 101 to 135 kb. All of these cases carried an identical ~1.2 Mb 4q32.2q32.3 duplication, which contained part of the *MARCH1* gene. *AFF2* gene duplications have been previously reported in the literature and have recently been implicated in auditory processing, emotional impairment and macrosomia [18]. The phenotypic spectrum of duplications of this gene (if any) is still not known. Three of our four cases exhibited phenotypes encompassing learning difficulties, microcephaly, developmental delay, dysmorphic features, and bilateral sensorineural hearing loss/audiology issues. The fourth case was part of a follow-up study for another CNV, but this patient was phenotypically normal. The DECIPHER database has a very small number of partial and intragenic *AFF2* gene duplications, but phenotypic information on most of these patients is not available. None of these cases had an additional 4q2.2q32.3 CNV. *MARCH1* is not covered by DGV, and it is unknown if the *MARCH1* duplication represents a “double hit” or exhibits a modifier effect on duplications of the *AFF2* gene.

Approximately 38 of the pathogenic X chromosome CNVs were aneuploid or large X chromosome abnormalities. Removal of these cytogenetically visible X chromosome rearrangements gives a detection frequency of 0.7 % for cryptic pathogenic CNVs, which is approximately double the frequency reported by Willemsen et al. [4] in their study of the clinical relevance of X chromosome CNVs. Their analysis was performed on patients with cognitive disorders, with or without congenital anomalies, and mainly using the Affymetrix® 250 k SNP array & Agilent 32 k BAC array. These workers suggested that relatively poor X chromosome coverage may have led to smaller X chromosome CNVs, such as those in the *MECP2* gene, being missed. The majority of our cryptic X chromosome CNVs ranged from 100 to 800 kb in size (including two cases of Xq28 duplication encompassing the *MECP2* and *L1CAM* genes). Of the remaining 37 cryptic pathogenic X chromosome CNVs, a small number of cases are of interest. Previously, *SHOX* gene deletions have been implicated in Léri-Weill dyschondrosteosis (OMIM # 127300) and Langer mesomelic dysplasia (OMIM # 249700) [19]. We found a small number of cases with agenic *SHOX* gene 3′ enhancer deletions which were initially not reported by our laboratory, but disruptions in this region are now known to be pathogenic [20]. We subsequently detected a 536Kb deletion in a patient with a complex phenotype including short stature and involving a deletion of the entire *SHOX* gene 3′ enhancer region. This represents the only agenic pathological CNV reported by our service to date. A small number of *SHOX* gene 5′ enhancer deleted CNVs have also been found; however, the significance of CNVs in this region has not been determined.

### Rare X chromosome variants

A blood sample was received of a 2 year old boy with a history of recurrent lymphadenitis, gastrointestinal involvement with mild inflammation of the stomach, with additional large intestine and perioral granulomatous disease. An immunology work up showed a provisional diagnosis of X-linked Chronic Granulomatous (OMIM # 306400). Microarray analysis showed a 109 kb deletion of the *CYBB* gene consistent with X-linked Chronic Granulomatous disease confirming the diagnosis. Finally a 1 year old boy who had global developmental delay carried a ~200 kb Xp21.3 deletion containing the *IL1RAPL1* gene, which is linked to non-specific X-linked mental retardation [21]. Subsequent testing showed that this deletion was inherited from his mother, as well as also being found in three of her unaffected sisters. Skewed X inactivation most likely accounted for phenotypic variability; however, further inheritance studies remain.

Both Coutton et al. [5] and D’Arrigo et al. [6] reported their microarray analysis of small cohorts of patients who had mild intellectual disability or developmental delay according to specific criteria. Their CNV detection frequency was approximately 30 %, with total pathogenic CNV yields of 16 and 21 %, and pathogenic X chromosome yields of 1.5 and 2.1 %, respectively. Despite the variation in sample sizes (60 vs 329), and geographic bias to one referral centre [6], a global CNV detection frequency of ~30 % is similar to our findings.

While specific phenotype studies have indicated a high detection frequency for pathogenic CNVs, a number of subsequent observations, including those reported here, report that ~9 % may be the common frequency for pathogenic CNVs detected in large scale heterogeneous populations. Deviations from this frequency may be due to the selection and enrichment of clinical phenotypes that may lead to the over-representation of common pathogenic regions such as those implicated in intellectual disability.

## Conclusion

Use of high resolution microarray platforms within our population has allowed the detection of pathogenic CNVs, as well as a significant number of cryptic pathogenic X chromosome CNVs. The latter may be due to a number of reasons. First, it represents a true reflection of the higher abnormality frequency in our population compared to others. Secondly, higher detection frequencies may be platform-specific, which allows for the detection of smaller pathological CNVs. Thirdly, we have been advantaged by the increased refinement of syndromic minimal critical regions and phenotypes, coupled with the discovery of additional new syndromes and pathological X-linked genes (such as *STAG2*). We consider the latter two reasons to be the most likely and so expect that detection frequencies of ~29 % for overall CNVs and ~9 % for pathogenic CNVs to be the minimum detection levels for laboratories. It is anticipated that additional larger studies in the future will match these detection frequencies, or possibly exceed them in the case of pathogenic X-linked disorders. The use of more targeted arrays in the future may be necessary to increase pathogenic detection frequencies. Our laboratory has recently implemented more rigorous triaging of referrals for microarrays, and also introduced the Affymetrix® Optima targeted array system as a replacement for BACs-on-Beads™ (BOBs) for the analysis of products of conception (POC). Both of these measures are expected to increase the detection of pathogenic CNVs.
